# Reinforcement Learning-Based Time-Slotted Protocol: A Reinforcement Learning Approach for Optimizing Long-Range Network Scalability

**DOI:** 10.3390/s25082420

**Published:** 2025-04-11

**Authors:** Nuha Alhattab, Fatma Bouabdallah, Enas F. Khairullah, Aishah Aseeri

**Affiliations:** 1 Faculty of Computing and Information Technology, King Abdulaziz University, Jeddah 21589, Saudi Arabia; nalhattab@stu.kau.edu.sa (N.A.); ekhairallah@kau.edu.sa (E.F.K.); aaaseeri@kau.edu.sa (A.A.); 2 Faculty of Science and Engineering, University of Plymouth, Drake Circus, Plymouth PL4 8AA, UK

**Keywords:** LoRa, Q-learning, LPWAN, scalability, IoT

## Abstract

The Internet of Things (IoT) is revolutionizing communication by connecting everyday objects to the Internet, enabling data exchange and automation. Low-Power Wide-Area networks (LPWANs) provide a wireless communication solution optimized for long-range, low-power IoT devices. LoRa is a prominent LPWAN technology; its ability to provide long-range, low-power wireless connectivity makes it ideal for IoT applications that cover large areas or where battery life is critical. Despite its advantages, LoRa uses a random access mode, which makes it susceptible to increased collisions as the network expands. In addition, the scalability of LoRa is affected by the distribution of its transmission parameters. This paper introduces a Reinforcement Learning-based Time-Slotted (RL-TS) LoRa protocol that incorporates a mechanism for distributing transmission parameters. It leverages a reinforcement learning algorithm, enabling nodes to autonomously select their time slots, thereby optimizing the allocation of transmission parameters and TDMA slots. To evaluate the effectiveness of our approach, we conduct simulations to assess the convergence speed of the reinforcement learning algorithm, as well as its impact on throughput and packet delivery ratio (PDR). The results demonstrate significant improvements, with PDR increasing from 0.45–0.85 in LoRa to 0.88–0.97 in RL-TS, and throughput rising from 80–150 packets to 156–172 packets. Additionally, RL-TS achieves 82% reduction in collisions compared to LoRa, highlighting its effectiveness in enhancing network performance. Moreover, a detailed comparison with conventional LoRa and other existing protocols is provided, highlighting the advantages of the proposed method.

## 1. Introduction

Low-Power Wide-Area networks (LPWANs) are wireless networks designed specifically for IoT devices. They enable long-range communication using minimal power, making them ideal for connecting devices in remote or hard-to-reach locations, such as rural areas or underground mines. LPWANs are also useful in applications where devices need to operate on battery power for extended periods. As the deployment of IoT devices continues to grow, LPWANs have become an essential component of the IoT ecosystem.

LoRa, which stands for Long-Range Wide-Area network, is a LPWAN technology designed specifically for IoT applications. It operates on unlicensed radio bands, making it cost-effective to deploy. By utilizing chirp spread spectrum modulation, it enables long-range communication with minimal power consumption, making it ideal for battery-operated devices [1]. LoRa employs a star-of-stars network architecture, where end-devices communicate with gateways that forward data to a centralized network server [2]. LoRa provides bi-directional communication, making it a versatile solution for various IoT applications, such as smart cities, agriculture, industrial monitoring, and asset tracking.

Although LoRa technology has been widely adopted in IoT applications, it faces several challenges that can affect its scalability and efficiency. One major issue is the use of the Aloha protocol for uplink communication. While this protocol allows devices to transmit data at any time, it can lead to collisions, particularly in densely populated environments where multiple devices may attempt to transmit simultaneously. Collisions between devices can lead to data loss and reduce the network efficiency.

Additionally, LoRa devices are subject to duty cycle restrictions imposed by regulations to prevent interference with other users of the unlicensed spectrum [1]. These restrictions limit the amount of time a device can transmit data within a given time window. The duty cycle compliance of LoRa networks can limit data throughput and real-time responsiveness, especially in applications that require frequent data transmission. Therefore, any attempt to optimally configure sensor nodes to improve network scalability and performance through a centralized solution running on the network server is compromised by the duty cycle restriction.

The distribution of transmission parameters is a critical issue in LoRa communication. Before transmission begins, several key parameters must be adjusted, including spreading factors, coding rates, transmission power, and channels. LoRa has six possible orthogonal spreading factors, ranging from SF7 to SF12, which are randomly selected by a sensor node. Notably, higher spreading factors increase communication range but also prolong transmission time. Additionally, LoRa supports multiple communication channels. For example, in Europe, there are typically eight available channels, each with a bandwidth of 125 kHz [3]. Choosing the most appropriate channel and spreading factor for communication helps reduce collisions. To mitigate collisions, simultaneous communications on the same channel must use different SFs, while simultaneous transmissions using the same SF must occur on different channels.

Furthermore, to enhance network scalability and flexibility, we aim to replace the Aloha medium access technique with a TDMA (Time Division Multiple Access-based approach. This introduces an additional level of separation, ensuring that nodes using the same spreading factor and channels are assigned different time slots to prevent collisions.

Reinforcement Learning (RL) can be applied in LoRa to optimize network performance and resource utilization. Using reinforcement learning (RL) to distribute TDMA slots presents an innovative approach to optimizing resource allocation in dynamic and decentralized environments. In traditional TDMA systems, slot allocation is typically predefined, making it less adaptable to varying network conditions, such as fluctuations in the number of devices or traffic patterns. By leveraging RL, the system can learn optimal slot assignments based on observed rewards, such as successful transmissions or minimized collisions. RL algorithms enable devices to dynamically adjust their behavior by exploring different slot allocations and exploiting those that maximize network performance. Moreover, RL can help address fairness concerns by ensuring that all devices have an equitable opportunity to access the medium over time.

The primary objective of our research is to mitigate collisions to the most possible extent. This can be achieved by determining the optimal distribution and assignment of LoRa transmission parameters, specifically channels, spreading factors, and time slots. By doing so, we can safely increase the number of nodes in the network, thereby enhancing the scalability of the LoRa network. Additionally, mitigating collisions can lead to further energy savings, which will also enhance the network’s lifetime. This work is applicable to LoRa-based IoT networks, where optimizing resource allocation and reducing collisions are critical. Potential applications include smart agriculture, smart cities, environmental monitoring, asset tracking, and industrial IoT, where reliable and scalable long-range communication with minimal power consumption is essential.

Our contributions can be summarized as follows.

- First, the introduction of a lightweight hybrid reinforcement learning (RL) solution that addresses the limitations of both fully centralized and fully decentralized RL approaches, particularly in the context of LoRa networks.
  Indeed, fully centralized RL approaches, while effective in leveraging global information, impose high computational burdens on the gateway and are severely constrained by duty cycle limitations. In large-scale, high-density networks, the time required to update nodes in duty-cycled environments becomes impractically long. On the other hand, fully decentralized RL approaches, though bypassing the duty cycle constraints, suffer from slow convergence as the nodes do not have full knowledge of the network conditions. Moreover, placing the entire computational load on energy-constrained sensor nodes can lead to significant resource exhaustion.
  To overcome these challenges, we propose a hybrid RL-based approach that achieves the following: (1) Minimizes the gateway’s role to simply broadcasting the level of congestion for each slot in the form of a compact vector, rather than performing complex calculations for every node to determine the optimal performance parameters. (2) Allows nodes to locally utilize this global congestion vector for fast convergence toward conflict-free, mutually exclusive slot assignments.
- Second, to achieve optimal overall configuration while respecting duty cycle constraints, our slot allocation mechanism is built on top of geographically based distributions of spreading factors (SFs), transmission powers (TPs), and channels. Unlike recent hybrid solutions in the literature, which attempt to optimize SF, TP, and channel selection simultaneously using RL—resulting in high computational overhead on resource-limited nodes—our approach statically assigns these parameters based on geographical positioning, as proposed in [4]. This enables RL to be focused solely on TDMA slot optimization, making the solution significantly more lightweight and scalable.

The rest of the paper is organized as follows: Section 2 reviews related work. Section 3 provides a detailed description of the protocol, including the mechanism used to set up the network field and an explanation of the learning algorithm. A comprehensive evaluation is then conducted in Section 4. Finally, we present our conclusions in Section 5.

## 2. Related Work

LoRa networks have gained prominence in IoT deployments due to their long-range communication, low power consumption, and scalability. However, their performance can be significantly impacted by the distribution of transmission parameters and the limitations of the ALOHA-based medium access control (MAC) protocol [5]. Optimizing these aspects has been a key focus of research to improve the reliability, scalability, and efficiency of LoRa networks.

### 2.1. Optimizing the Distribution of Transmission Parameters

The proper allocation of transmission parameters, such as spreading factors (SFs), power levels, and channels, is crucial for maximizing network efficiency. Gkotsiopoulos et al. [6] conducted a comprehensive review highlighting the performance determinants of LoRa networks. Their study emphasized the importance of balancing SF distribution to ensure equitable resource utilization and minimize collisions. Unoptimized SF allocation can lead to channel congestion and inefficient spectrum usage, particularly in dense deployments. Zorbas et al. [7] explored a method to optimize LoRa’s capacity through multiple spreading factor configurations. Their approach dynamically adjusts SFs based on traffic conditions and channel quality, improving spectral efficiency and reducing interference. While their method significantly enhances throughput and scalability, the dynamic adjustment of spreading factors may pose challenges in managing heterogeneous devices with varying capabilities, especially in large-scale deployments. Reynders et al. [8] introduced a lightweight scheduling mechanism to improve the reliability and scalability of LoRaWAN. Instead of assigning specific transmission parameters to individual nodes, their approach schedules channels by dynamically specifying the allowed transmission powers and spreading factors for each subframe. Nodes then independently select their transmission parameters, including transmission power, SF, and channel, based on this high-level scheduling information. Within these subframes, nodes utilize random access, maintaining an ALOHA-like behavior. This structured randomness enhances channel utilization and provides a low-overhead solution, making it suitable for large-scale deployments. However, the simplicity of the method may limit its adaptability, as random access within subframes can still result in collisions, particularly when the network conditions are dynamic or unpredictable. Gopal and Prabhaka [9] proposed a hybrid approach to transmission parameter optimization, leveraging centralized coordination for efficient SF and power level allocation. By combining global insights with local adaptability, their method ensures optimal parameter distribution while maintaining computational efficiency. This approach effectively addresses the scalability challenges associated with purely centralized systems.

### 2.2. Time Slot-Based Solutions

Several studies have proposed replacing ALOHA with time-slotted MAC protocols to improve reliability and network performance [10,11]. Zorbas et al. [12] developed TS-LoRa, a time-slotted LoRaWAN protocol tailored for industrial IoT (IIoT) environments. TS-LoRa replaces ALOHA with deterministic time-slot allocations, providing predictable latency and eliminating collisions. This makes it highly suitable for applications requiring strict timing constraints. However, the rigid structure of TS-LoRa may reduce flexibility in networks with irregular traffic patterns or varying device activity. Similarly, Alahmadi et al. [13] proposed a novel time-slotted MAC protocol to enhance scalability in IoT networks. Their protocol incorporates advanced slot allocation mechanisms to minimize collisions and improve throughput. By optimizing slot usage, this method achieves superior performance in terms of packet delivery ratio (PDR) and throughput.

However, in time-slotted protocols like those proposed by Alahmadi et al. [13] or Zorbas et al. [12], the allocation of time slots often requires coordination and scheduling, which introduces a computational burden. The system may need to assign slots dynamically based on traffic patterns, device priorities, or other network conditions. This process involves tracking device states, optimizing slot usage, and possibly adapting schedules in real time. LoRa networks often operate with devices that are power-limited and computationally constrained. These devices may struggle to handle complex slot allocation algorithms, especially if the allocation requires frequent updates or involves significant processing at the device level.

### 2.3. RL-Based Solutions

Reinforcement learning (RL) has been widely employed in LoRa networks to optimize medium access control (MAC) protocols and resource allocation [14,15]. Reinforcement learning can significantly reduce the computational burden associated with traditional slot allocation mechanisms. This section reviews relevant studies, which can be broadly categorized into centralized, decentralized, and hybrid learning approaches, each offering unique advantages and challenges.

#### 2.3.1. Centralized Approaches

Centralized RL approaches depend on a single controller to manage the learning process for all nodes in the network. This controller collects information from the whole network, trains the RL model, and distributes actions or policies to individual nodes. Such methods ensure globally optimal solutions but may face scalability and communication overhead challenges, especially in duty-cycled networks. For example, H. Zhong et al. [16] employed a centralized deep reinforcement learning (DRL) approach to optimize the allocation of spreading factors (SFs) in LoRa networks, improving throughput and spectral efficiency by leveraging global traffic and channel state information. Similarly, R. Hamdi et al. [17] proposed LoRa-RL, a DRL-based framework for managing resources in hybrid energy LoRa networks, achieving efficient energy and resource allocation. Sandoval et al. in [18] proposed a mathematical optimization model to derive the best transmission configurations for all nodes and then reinforcement learning is used at the gateway to determine optimal update policies to disseminate these configurations to nodes. However, the reliance on a central controller in such approaches makes them susceptible to bottlenecks and increases communication overhead in large-scale duty-cycled networks.

#### 2.3.2. Decentralized Approaches

Decentralized RL enables individual nodes to learn their policies independently based on local observations and rewards. This approach offers greater scalability and robustness to failures, as nodes do not rely on a central controller. However, the lack of global coordination often results in slower convergence and suboptimal performance, particularly in dense networks or large-scale deployments.

For instance, T. Onishi et al. [19] proposed an RL-based collision avoidance mechanism for distributed IoT systems, where each node acted as an independent agent, reducing collisions and improving network reliability. Similarly, X. Huang et al. [20] developed a decentralized RL-based MAC protocol for LoRa networks, allowing nodes to independently adjust their transmission strategies to minimize collisions and enhance throughput. While both approaches demonstrated adaptability to local conditions, their decentralized nature limited optimization in dense or dynamic environments. A. Ivoghlian et al. [21] extended this concept by employing a multi-agent RL framework that enabled collaborative optimization of transmission parameters among nodes. This introduced some level of cooperation, improving scalability and robustness, yet the reliance on local observations still constrained the ability to achieve globally optimal solutions.

#### 2.3.3. Hybrid Approaches

Hybrid learning solutions emerged in an attempt to combine the advantages of both centralized and decentralized approaches. This approach uses partial global information to guide decisions while maintaining local adaptability. Hybrid RL is particularly effective in scenarios where full centralization is impractical due to scalability constraints, while full decentralization is insufficient to ensure optimal performance.

Running optimization algorithms on the sensor nodes would drain their limited battery life, as machine learning models require high computational and energy. To mitigate this issue, Zhao et al. in [22] proposed a Multi-Agent Reinforcement Learning (MARL) algorithm that utilizes a Centralized Training with Decentralized Execution (CTDE) approach. This means that all the computational work is handled by the gateway. While the gateway centrally collects the states of all nodes, it allocates computational resources for each agent corresponding to a node, so each agent assigns a reward function based on its impact on the overall performance. In their work, they focused on adjusting the spreading factor (SF) and transmission power (TP) to improve transmission quality and energy efficiency in the LoRaWAN-based underground network. The introduced MARL-based algorithm effectively reduced collisions and improved the scalability of the network. Similarly, G. Park et al. in [23] proposed a hybrid learning approach for network resource optimization, specifically optimizing the distribution of spreading factors, transmission powers, and channels. The gateway gathers network state information which allows a global view of the network, while each LoRa node has an associated RL agent that learns independently at the network server; after training, each node follows its own learned policy. Note that, instead of assigning one RL agent per node, nodes at similar distances from the gateway are grouped and managed by a single RL agent. This method balances global coordination with local flexibility, improving network efficiency while mitigating the overhead associated with centralized approaches.

This article presents a hybrid RL-TDMA-based approach to address the limitations of existing solutions. Table 1 presents a comparison between RL-TS and the previously discussed works. The proposed protocol allows nodes to (1) independently determine their spreading factors and channels based on their geographical coordinates, and (2) autonomously select their time slots using a hybrid RL algorithm. The proposed protocol combines the coordination strengths of centralized systems with the lightweight scalability of decentralized systems. Detailed explanations are provided in the following section.

## 3. Protocol Description

### 3.1. Setting the Environment

**Figure 1:**
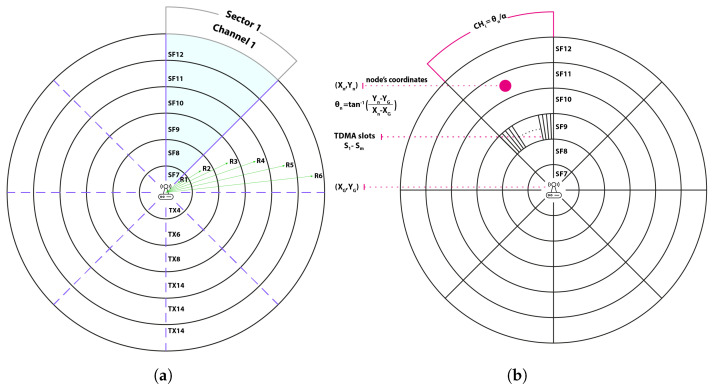
Network partitioning. (**a**) SFs among cells and channels among sectors (**b**) TDMA slots among cell–sector intersection.

In this paper, we propose a distributed protocol where nodes autonomously select their SFs, communication channels, and TDMA slot identifiers based on their relative positions from the gateway. Building on the network architecture provided by [4], the network field is divided into six cells, each assigned a specific spreading factor and transmission power, starting from the lowest values. Consequently, SF7, with the lowest transmission power, is used by nodes in the closest cell to the gateway, followed by SF8 with the second-lowest transmission power, and so on, as shown in Figure 1a. As shown in Table 1, each LoRa SF has a specific range, where larger SFs are exclusively used for greater distances from the gateway to ensure successful packet delivery. Thus, similar to the approach proposed in [4], each node autonomously determines its SF based on its distance from the gateway using Table 2 [24].

For instance, if a node *n* has a distance $d_{n}$ of 3 km from the gateway, SF8 will be selected. In other words, if $r_{i-1}<d_{n}\leq r_{i}$, the SF of cell *i* will be selected, where $r_{i}$ is the range of cell *i*. Regarding channel distribution, we further divide the field into eight sectors so that nodes in each sector will be assigned a specific channel for communication as shown in Figure 1a. In order for a node *n* to autonomously select its channel without requiring any communication with the server, it will start by calculating its polar angle $\theta_{n}$ from the gateway using Equation (1)

(1) $$\theta_{n}=tan^{-1}\left(\frac{Y_{n}-Y_{G}}{X_{n}-X_{G}}\right)$$

where $\left(X_{n},Y_{n}\right)$ are the geographical coordinates of node *n*, and $\left(X_{G},Y_{G}\right)$ are those of the gateway. Once $\theta_{n}$ is derived, node *n* determines the sector identifier to which it belongs, which also serves as the channel identifier, using Equation (2):

(2) $$CH_{n}=\frac{\theta_{n}}{\alpha }$$

where α=π/8, as eight channels are available in the European ISM band—see Figure 1b. Note that nodes in the same sector and the same cell use the same channel and spreading factor. Therefore, collisions may occur among these nodes. To mitigate such collisions, we employ Time Division Multiple Access (TDMA) as the medium access technique; see Figure 1b. Accordingly, each node is assigned a predetermined slot identifier for communication. The total number of required slots *m* in a given cell-sector intersection is derived using Equation (3):

(3) $$m=N_{d}\cdot \frac{\alpha }{2}\left(r_{i}^{2}-r_{i-1}^{2}\right)$$

where $N_{d}$ is the network density. Note that the network density $N_{d}$ can be derived using Equation (4):

(4) $$N_{d}=\frac{N}{\pi \cdot R^{2}}$$

where *N* is the total number of nodes in the network and *R* is the field radius. To ensure conflict-free slot allocation among nodes, we distribute the TDMA slots among nodes based on a reinforcement learning algorithm, specifically Q-learning.

### 3.2. Learning Algorithm

Q-learning, a cornerstone of reinforcement learning, addresses the challenge of decision-making in sequential environments. At its core are several components that drive its operation. First, states (s) embody the different configurations that an agent encounters while interacting with its environment. Actions (a) denote the choices available to the agent in each state, shaping its path through the environment. Central to Q-learning are Q-values (Q(s,a)), which represent the expected cumulative rewards of taking an action from a given state and subsequently following an optimal policy. Rewards (r) serve as immediate feedback, quantifying the immediate benefit or cost of an action. Meanwhile, the discount factor (γ) weighs future rewards against immediate ones, influencing the agent’s preference for short-term gains over long-term benefits. Together, these components orchestrate the process of learning optimal policies in uncertain environments, guiding the agent’s actions toward maximizing cumulative rewards. Equation (5) is for updating the Q-value, which is at the core of Q-learning [25].

(5) $$Q\left(s,a\right)\leftarrow Q\left(s,a\right)+\alpha [r+\gamma \max_{a^{\prime }}Q\left(s^{\prime },a^{\prime }\right)-Q\left(s,a\right)]$$

where

- Q(s,a) is the current Q-value of the state–action pair.
- α is the learning rate, determining the extent to which newly acquired information overrides old information. A smaller learning rate leads to slower convergence but more stable learning.
- *r* is the immediate reward obtained after taking action a in state s.
- γ is the discount factor, as described earlier.
- $max_{a}^{\prime }Q\left(s^{\prime },a^{\prime }\right)$ represents the maximum Q-value achievable from the next state considering all possible actions $a^{\prime }$.
- $s^{\prime }$ is the state reached after taking action *a* from state *s*.

Equation (5) updates the Q-value of the current state–action pair based on the observed immediate reward and the estimated future rewards. Over time, as the agent explores the environment and receives feedback, the Q-values converge to accurately represent the expected cumulative rewards for each action in each state, enabling the agent to make informed decisions and learn an optimal policy.

Multi-agent Q-learning extends the principles of Q-learning to scenarios where multiple agents interact within the same environment, each making decisions independently to achieve individual or collective goals. Since we adopt multi-agent Q-learning, in our protocol, both the states and actions correspond to the TDMA slots available for a given cell-sector intersection. The agents are the sensor nodes, where learning is applied to optimize the distribution of TDMA slots among them, to ensure conflict-free slot assignments.

As mentioned earlier, each agent is assigned a predefined SF and a given channel based on its relative position and polar angle from the gateway, respectively. In the first round, each agent randomly selects its state (current TDMA slot) from the available slots. We then apply a collision detection algorithm that passes collision values to a function called computeReward(). The collision detection algorithm is described in the following section. The computeReward function utilizes the collision information to allow each agent to calculate its own reward value. Table 3 outlines the reward assignment procedure for nodes within a cell–sector intersection. Accordingly, each node that does not experience any collision on its selected slot assigns itself a high reward for that slot. However, if a collision occurs, rewards are assigned to slots based on the number of collisions experienced. Specifically, first, a node *n* assigns itself a negative reward for any slot selected by other nodes if they experienced no collisions on their chosen slots. By doing so, a node aims at discouraging itself from selecting slots where other nodes have transmitted without collisions. Second, node *n* assigns itself a high reward for any slot that has not been selected by any other node. This encourages the node to switch to non-selected slots. Third, node *n* assigns itself a non-zero reward for any slot that has experienced collisions. The assigned reward is inversely proportional to the number of collisions experienced on the slot. Once rewards for the slots are locally assigned by each agent, every agent applies a reinforcement learning algorithm to determine its next state (next TDMA slot) by calculating and updating Q-values in its own Q-table, using the update Equation (5) mentioned earlier.

Please note that after each round of transmission, the gateway will send a beacon message containing the number of experienced collisions in each slot as a broadcast message to nodes in the same cell–sector intersection. It is worth noting that, in order to minimize the beacon message size, non-selected slots will not be mentioned in the beacon message. In case all the slots are selected, the ones with zero collisions will not be mentioned.

### 3.3. Collision Detection Algorithm

Multi-agent learning algorithms can be broadly classified into two approaches: centralized and decentralized. In the centralized approach, agents benefit from a global view of the environment, enabling more efficient conflict resolution and faster convergence. However, this approach faces challenges in terms of scalability and incurs high communication and computational costs as the number of agents increases. In contrast, a decentralized approach allows each agent to learn independently based on its local observations and reward signals. This improves scalability and resilience to failures. However, decentralized systems often exhibit slower convergence and suboptimal performance due to the absence of global coordination. In dense networks, this lack of coordination can lead to increased collisions and reduced throughput, further limiting efficiency. In this study, we propose a hybrid approach that combines the strengths of both centralized and decentralized multi-agent learning. Our proposed method leverages the global view and coordination capabilities of a centralized system to optimize decision-making while integrating the lightweight and scalable nature of decentralized systems to reduce computational overhead and enhance adaptability. In our collision detection algorithm, we assign a value to each node–slot pair as follows: an unused slot receives a value of −3, a successful transmission in a slot receives a value of 0, and for each collision detected on a slot, its value is incremented by 1. At the end of all transmissions, we consider only the highest observed value for each slot, forming a vector of size equal to the number of slots. This vector provides a comprehensive global view that nodes can leverage while maintaining a compact size, consistent with the decentralized approach.

**Figure 2:**
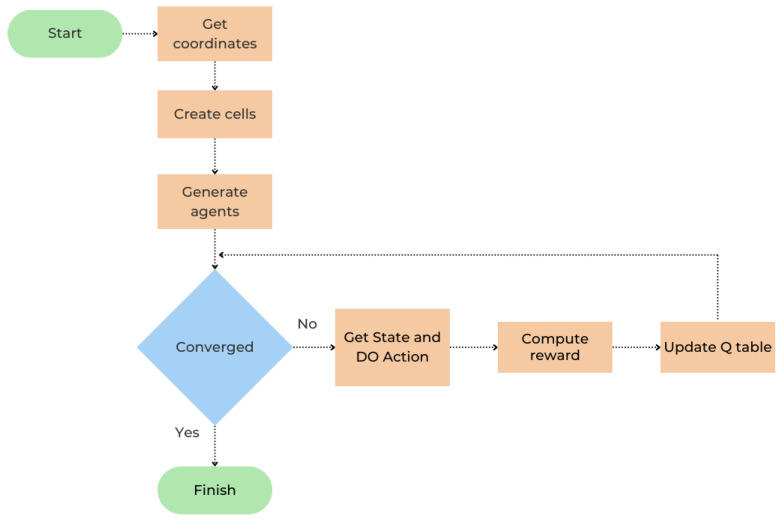
Flowchart of RL-TS protocol.

The flowchart in Figure 2 describes the entire process of our protocol. After dividing the network field into cells and sectors based on the geographical coordinates of the gateway, agents are created and associated with each sensor, each with its associated SF and channel. Agents autonomously select their SFs and channels based on their distances from the gateway (as described earlier in Section 3.1). The agents then undergo a parallel learning process with the common goal of reaching convergence; the learning process and the compute reward algorithm are described in Section 3.2. Once each agent at a cell–sector intersection is assigned its own mutually exclusive TDMA slot, the network reaches convergence, and the learning process is completed. Thereby, the learning process is complete, ensuring conflict-free communication.

## 4. Performance Evaluation

In this section, we present the results of a comprehensive simulation-based evaluation aimed at assessing the performance, efficiency, and robustness of our proposed protocol using Matlab R2024b. Matlab is a widely used software platform for research, development, and implementation of various machine learning algorithms. It provides a versatile programming environment that supports the implementation of various RL algorithms such as Q-learning. First, we describe the parameter settings, followed by an evaluation of the performance of our Q-learning algorithm in two test cases. This assessment is based on the following performance criteria: number of required episodes (time to convergence), number of collisions, throughput, and Packet Delivery Ratio (PDR) until convergence. Please note that Equation (6) and Equation (7) are used to calculate the PDR and the throughput, respectively.

(6) $$PDR=\frac{SuccessfullyReceivedPackets}{TotalPacketSent}$$

(7) $$Throughput=\frac{SuccessfullyReceivedPackets\times PacketSize}{SimulationTime}$$

### 4.1. Parameter Settings

Since our model is designed for large networks with high node connectivity, the randomly selected cell–sector intersection includes connected nodes ranging from 20 to 200. We have defined a class that encapsulates the properties of the nodes, including ID, channel, SF, time, reward, and Q-table. In addition, the class contains functions such as computeReward() and reinforcementLearning(), ensuring that each node acts as an agent, instantiated as an instance of this class. Table 4 presents the parameter settings for the evaluation tests.

### 4.2. RL-TS Evaluation Results

#### 4.2.1. Q-Learning Algorithm Evaluation

**Figure 3:**
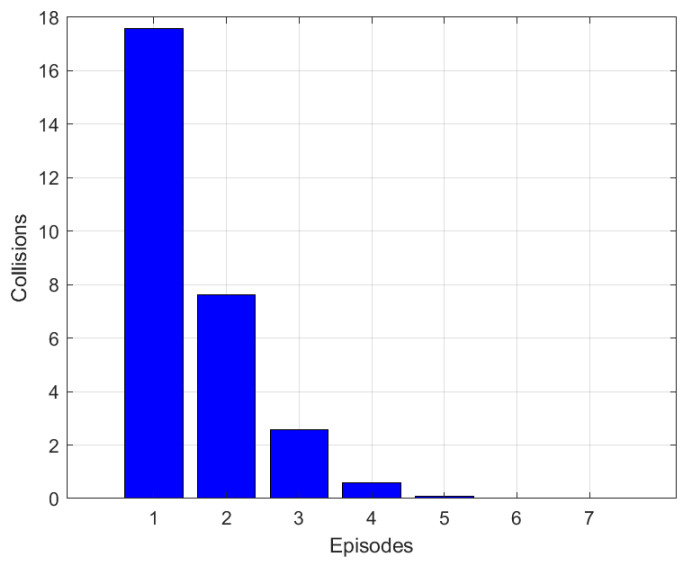
Number of collisions over episodes until convergence.

To evaluate the speed of our Q-learning algorithm, we tested the average changes in the number of collisions over episodes until convergence. This test was conducted with 60 nodes and 80 TDMA slots. As shown in Figure 3, there was a steep drop in the number of collisions from the second episode, and it continued to decrease over subsequent episodes, demonstrating the robustness and efficiency of our proposed Q- learning algorithm. Notably, starting from episode 4, fewer than 1 collision occurs across the entire network. Similarly, the PDR achieved at convergence is equal to 0.93, which highlights the effectiveness of our conceived algorithm, especially the computeReward() function explained in Table 3.

#### 4.2.2. Case 1: The Number of Available TDMA Slots Equals the Number of Nodes

**Figure 4:**
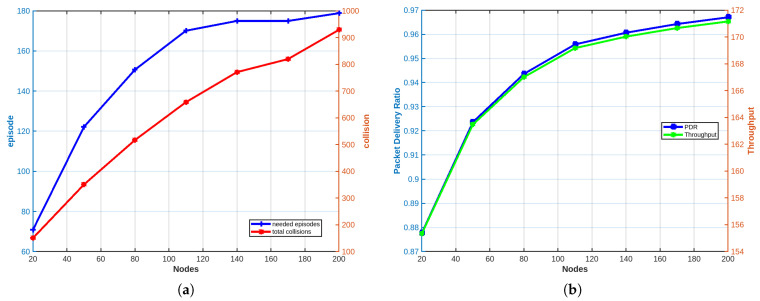
Case 1: (**a**) Convergence time and total collisions results. (**b**) PDR and throughput results.

In this case, we assume an ideal scenario where the number of TDMA slots is equal to the number of nodes in a given cell–sector intersection. As shown in Figure 4a, as the number of nodes increases, more collisions occur until convergence. In addition, the number of episodes required to reach convergence, where each node is assigned its own slot, also increases. Figure 4b illustrates an increase in throughput and packet delivery ratio (PDR) as the number of nodes grows. Since the number of available slots matches the number of nodes, our adopted Q-learning algorithm ensures that as the number of nodes increases, the number of successfully received packets per episode grows faster than the number of collisions. Consequently, a clear increase in PDR and throughput is observed, as depicted in Figure 4b. It is worth noting that PDR values range between 0.87 and 0.96, underscoring the robustness of our Q-learning algorithm, particularly the computeReward() function. Scalability refers to a network’s ability to accommodate increasing demands while maintaining performance levels. The observed high throughput suggests that the network can effectively scale to handle larger workloads without compromising efficiency. Furthermore, the high PDR underscores the reliability of packet transmission, ensuring a significant proportion of packets successfully reaching their intended destination.

#### 4.2.3. Case 2: Constant Number of Available TDMA Slots

**Figure 5:**
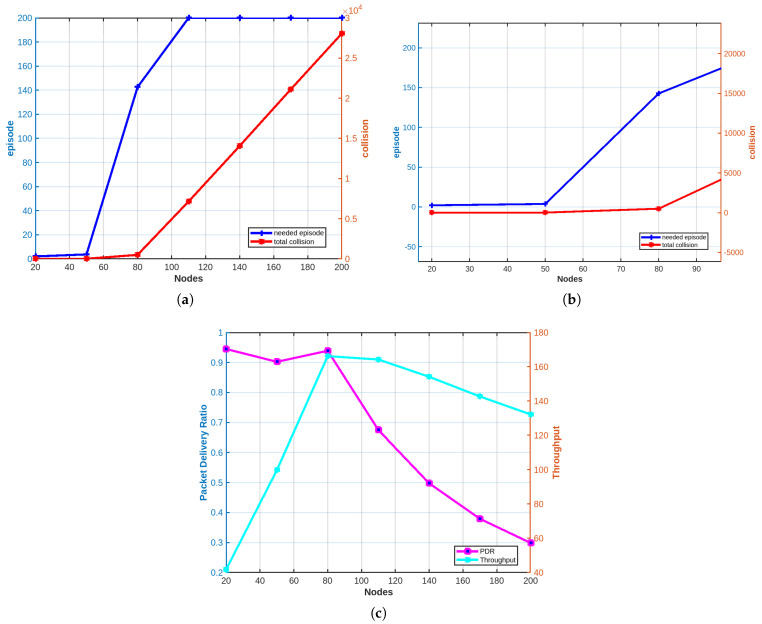
Case 2: (**a**) Convergence time and total collisions results. (**b**) A closer view. (**c**) PDR and throughput results.

In this case, we evaluate the network performance when the number of TDMA slots is fixed, regardless of the number of nodes. Recall that since nodes are randomly distributed in the field, using Equation (3) to calculate the required number of slots may lead to either overestimation or underestimation of the number of required slots. Hence, the rationale behind setting a fixed number of TDMA slots. In our experiment, we set the number of available slots to 80. From Figure 5a,b, we can observe that as the number of nodes competing for the TDMA slots increases, both the number of collisions and the time to convergence also increase. Notably, when the number of nodes exceeds 80, which is the number of available slots, the number of collisions and the number of episodes will highly increase as the number of nodes is greater than the number of available slots and hence convergence will never be reached. In Figure 5c, when the number of nodes is less than the number of available slots, a local minimum occurs at 50 nodes, while a global maximum is achieved with 80 nodes. This behavior results from both the increase in collisions (as shown in Figure 5a) and the increase in the number of nodes. Recall that according to our algorithm, in each episode, all nodes transmit. Thus, increasing the number of nodes inevitably increases the number of transmissions. The compromise between the rise in collisions and the increase in transmissions leads to the creation of local and global optimums. However, when the number of nodes exceeds the number of available slots, the PDR decreases, and the throughput stabilizes. For example, when the number of nodes in a cell–sector intersection reaches 200, the achieved PDR equals only 0.4. In this case, the 80 available slots are 2.5 times less than the number of nodes. Thus, the probability that the network will undergo a collision is equal to 1. The stability in throughput immediately after the number of nodes matches the number of available slots is due to network saturation, where the number of successfully received packets no longer increases due to collisions. Thus, we can conclude that carefully designing the total number of slots m will be the focus of our future work, as our conceived algorithm is sensitive to m. In fact, setting the number of slots equal to the number of nodes is more efficient, as it optimizes both the PDR and the throughput, thereby achieving better scalability.

### 4.3. Comparison

To further evaluate RL-TS, we compare it with different protocols, namely LoRa, the RL-based MAC protocol presented in [20], and RL-TS-Decentralized.

#### 4.3.1. Comparing with LoRa

**Figure 6:**
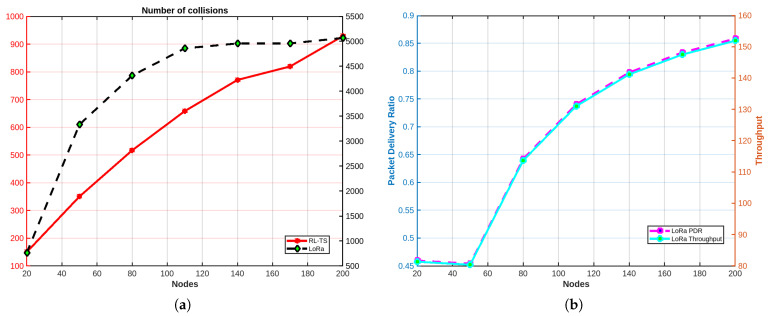
Case 1 (**a**) total collisions for both protocols. (**b**) LoRa performance.

**Figure 7:**
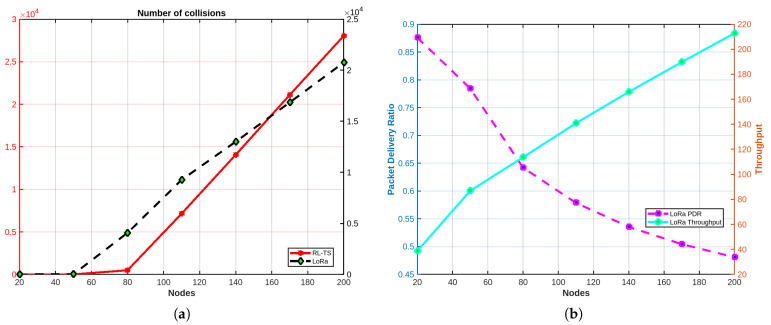
Case 2: (**a**) Total collisions for both protocols. (**b**) LoRa performance.

LoRa uses Aloha as a contention-based protocol, allowing devices to transmit whenever ready, which leads to frequent collisions, particularly in dense networks. In contrast, the RL based TDMA protocol used by RL-TS assigns predefined time slots to devices, effectively eliminating collisions by ensuring non-overlapping transmissions. To ensure a fair and unbiased comparison between RL-TS and LoRa, we accounted for the number of episodes required for our protocol to converge. Specifically, the LoRa evaluation was conducted over an equivalent number of episodes as required by RL-TS to reach convergence. Additionally, the time duration for LoRa was calculated based on the equivalent number of TDMA slots. This approach ensures that both protocols were assessed under comparable conditions, allowing an accurate evaluation of their performance metrics, including collisions, packet delivery ratio (PDR), and throughput. The results show that RL-TS achieves a substantial reduction in the number of collisions compared to LoRa, significantly improving network reliability. Moreover, RL-TS demonstrates a notable improvement in packet delivery ratio (PDR) and throughput, as it maximizes the utilization of available time slots and ensures that more packets are successfully delivered. In contrast, LoRa’s performance in terms of PDR and throughput is hindered by the overhead of retransmissions and wasted resources caused by collisions. These findings emphasize the superiority of our protocol in managing medium access for LoRa networks, particularly in scenarios demanding high reliability, efficiency, and scalability. Figure 6 and Figure 7 show the evaluation results of LoRa and RL-TS for case 1 and case 2, respectively.

#### 4.3.2. Comparing with RL-Based Protocol by X. Huang et al.

X. Huang et al. in [20] use the Policy Hill Climbing (PHC) algorithm as a reinforcement learning technique used in multi-agent systems to iteratively improve an agent’s policy based on observed rewards. PHC combines traditional Q-learning with probabilistic policy updates. In each iteration, the agent selects an action based on its current policy, observes a reward, and updates its Q-values using the Bellman equation. The policy is then adjusted by favoring the action with the highest Q-value, while gradually reducing the probabilities of other actions. The update rule ensures smooth policy adjustment, balancing exploration and exploitation, and is often controlled by parameters such as learning rate (α) and policy adjustment factor (δ). This approach enables agents to adapt dynamically to changing environments, making it suitable for complex scenarios such as medium access control in networks.

**Figure 8:**
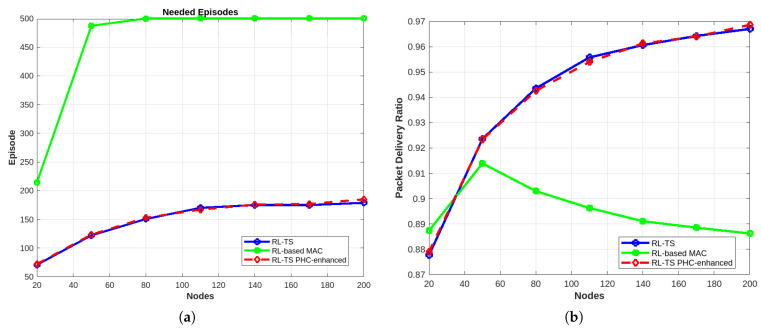
(**a**) Convergence time results. (**b**) PDR results.

A comparison is conducted between RL-TS and the RL-based MAC protocol presented in [20]. This comparison aims to evaluate the performance of RL-TS against the RL-based protocol, which employs the PHC algorithm to improve multi-access efficiency, in terms of Packet Delivery Ratio (PDR), collision rates, and scalability in dense network environments. This comparison evaluates both protocols in scenarios with varying device densities to determine their relative effectiveness in minimizing collisions and maximizing PDR. While the RL-based MAC protocol proposed in [20] demonstrates robust performance in moderate network conditions, its fully decentralized nature, combined with reliance on probabilistic action selection, can lead to instability and inefficiencies in highly dense networks. Figure 8a shows that as the number of devices increases, the convergence time increases significantly in the RL-based MAC protocol, “and sometimes the system fails to converge even after 2000 episodes” [20]. In contrast, the RL-TS demonstrates superior efficiency, achieving convergence in less than 200 episodes even in the densest network scenarios. As shown in Figure 8b, it is evident that the RL-based MAC protocol reaches its peak performance at approximately 40 devices, after which the performance begins to degrade.

To further evaluate the efficiency of our proposed protocol, we compared it with an enhanced version using the PHC algorithm; we call it RL-TS PHC-enhanced. In this version, we propose an improvement in the calculation of the policy in the PHC algorithm as follows. Initially, the policy is uniformly initialized, with equal probabilities assigned to each action based on the number of available slots. As rewards are observed, the algorithm updates the policy for actions that yield positive rewards by normalizing each action’s reward against the sum of all positive rewards using Equation (8). This ensures that actions with higher rewards receive higher probabilities, as described in the pseudocode shown in Algorithm 1.

(8) $$\pi \left(s,a\right)=\frac{R(s,a)}{\sum_{a^{\prime }\in A}R\left(s,a^{\prime }\right)}$$

where the following hold:

- π(s,a): The probability of selecting action a in state s.
- R(s,a): The reward for taking action a in state s.
- $\sum_{a^{\prime }\in A}R\left(s,a^{\prime }\right)$: The total reward for all actions a′ in state s.
- *A*: The set of all actions in state *s*.

**Algorithm 1** RL-TS PHC-enhanced  1:Initialize $\pi =\frac{1}{\text{number\_of\_slots}}$ for all actions a∈A  2:**for** each action *a* **do**$$\pi \left(a\right)=\left\{\begin{array}{cc} \frac{\mathrm{reward}(a)}{\sum_{a^{\prime }\in A}\mathrm{reward}\left(a^{\prime }\right)}, & \mathrm{if}\;\mathrm{reward}(a)>0\\ 0, & \mathrm{otherwise} \end{array}\right.$$  3:**end for**  4:Set η←0.8  5:n←COUNT_NON_ZERO(π)  6:**for** each action a∈n **do**  7:   $\eta \left(a\right)\leftarrow \min(\pi \left(a\right),\frac{\eta }{n})$  8:**end for**  9:$\mathrm{TD}\leftarrow \mathrm{reward}(a_{t},a_{t+1})+\gamma \cdot \pi (a_{t},a_{t+1})\cdot Q(a_{t+1},\mathrm{argmax})-Q(a_{t},a_{t+1})$10:**Update *Q* using Bellman equation**11:$\pi (a_{t},a_{t+1})\leftarrow \pi (a_{t},a_{t+1})+\Delta \cdot \pi (a_{t},a_{t+1})$$$\Delta =\left\{\begin{array}{cc} -\eta (\mathrm{current},\mathrm{next}), & \mathrm{if}\;a_{t+1}\neq argmax\\ \sum_{a^{\prime }\in A}\eta \left(a_{t},a^{\prime }\right), & \mathrm{otherwise} \end{array}\right.$$

The comparison aims to examine the differences in performance metrics while also considering the computational overhead introduced by PHC. The results show that both protocols achieve comparable performance in key metrics such as packet delivery ratio (PDR), throughput, and convergence time, with only slight differences observed, as shown in Figure 8. However, in terms of computational overhead, our protocol demonstrates a significant advantage. Although frequent updates to the policy and the calculation of η can become computationally expensive in environments with a large action space, our protocol remains lightweight and simpler to implement. This makes it more suitable for resource-constrained devices commonly used in LoRa networks. By avoiding the computational overhead of PHC while maintaining comparable performance, our protocol presents a more practical solution for real-world deployments.

#### 4.3.3. Decentralized Multi-Agent Learning

**Figure 9:**
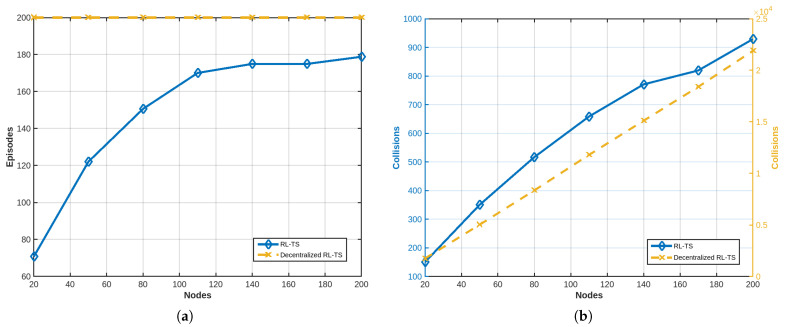
Results for decentralized RL-TS. (**a**) Number of needed episodes compared to RL-TS. (**b**) Number of collisions compared to RL-TS.

As mentioned earlier, our proposed method leverages the global view and coordination capabilities of a centralized system to optimize decision-making, while integrating the lightweight and scalable nature of decentralized systems to reduce computational overhead and improve adaptability. By striking a balance between the two paradigms, our approach maintains high performance in terms of convergence, throughput, and packet delivery ratio (PDR). This hybrid framework ensures efficient resource allocation and robustness, making it particularly suitable for dynamic and resource-constrained environments of LoRa networks. As shown in Figure 9, the decentralized protocol failed to converge, even in a non-crowded network, resulting in very high collisions.

## 5. Conclusions

This paper introduced a novel RL-based TDMA medium access protocol for LoRa networks, replacing the existing random access protocol, i.e., Aloha. Traditional LoRa networks suffer from performance degradation as the number of devices increases due to interference, collisions, and inefficient parameter allocation. In this study, we introduced a lightweight approach, where SFs, channels, and transmission powers are statically assigned as proposed in [4], while RL is used solely to optimize TDMA slot allocation. By doing so, we first ensure that devices using the same SFs are portioned by channel allocation to avoid collisions. Nodes sharing the same SFs on the same channel are then further separated through RL-based slot assignment. Consequently, three levels of separation are employed to ensure collision-free communication among nodes. This allows high-density networks to be safely handled as resources are shared more efficiently, thus enhancing network capacity and scalability. Accordingly, collision-free communication is guaranteed for any network density, provided that enough slots are available. Second, we ensure that scalability is improved without overwhelming the network’s computational and management capabilities, particularly on the node side. SFs, channels, and transmission powers are allocated statically based on the node’s geographical coordinates relative to the gateway, thereby avoiding communication overhead between the gateway and the nodes for transmission parameter selection. As for our RL-based slot assignment, it is hybrid and lightweight and thus can easily adapt to large-scale networks without generating significant computational overhead. Its effectiveness was demonstrated through simulation-based evaluation, especially when the number of available slots was equal to the number of nodes in a cell–sector intersection. In this case, our protocol resulted in an 82% reduction in collision rates over the episodes, compared to LoRa. Indeed, using RL for slots optimization significantly improved packet delivery ratio (PDR), maintaining it within the 0.88–0.97 range across various network densities. This improvement reduced retransmissions, ultimately leading to higher throughput and improved scalability as well as energy efficiency.

That being said, accurately estimating the number of slots required for efficient communication can be a challenge. While we propose a formula to determine the required number of slots, variations in node distribution patterns may lead to underestimation or overestimation of required slots. Thus, dynamically adjusting the required number of slots will be of great help when it comes to further improving the network performance in real-world scenarios.

## Figures and Tables

**Table 1:** Comparing RL-TS with existing works.

Ref.	Methodology	Advantages	Limitation	Comparison with RL-TS
[12]	Deterministic time slot allocation	Predictable latency, no collisions	Less flexibility for variable traffic loads	RL-based time slot allocation
[13]	Divides network into cells, sub-cells, and sectors for sector-based time slot allocation	Reduces collisions, improves PDR and throughput	High coordination and scheduling overhead	Nodes autonomously select their SFs and channels based on geographical coordination
[16,17,18]	Using RL at gateway for resource allocation	Improves spectral efficiency and throughput	High communication overhead	Using RL at nodes for slot allocation
[19,20,21]	Collaborative learning at end devices	Reduces collisions, improves robustness	Slow convergence	Hybrid approach for faster convergence
[22,23]	Using hybrid RL for SFs, TP and channel optimization	Reduces collisions, improves scalability	High computational overhead on energy constrained nodes	Geographical-based allocation of SF, TP, and channels, using hybrid RL for slot allocation

**Table 2:** Spreading factor ranges [24].

SF	Range
SF 12	12 km
SF 11	10 km
SF 10	8 km
SF 9	6 km
SF 8	4 km
SF 7	2 km

**Table 3:** Reward assignment algorithm.

Step	Description
1. Input	Node n∈N, current time slot $t_{c}\in T$, maxCollision[T].
2. Output	Reward(n,T).
3. Check collisions at $t_{c}$	If $\mathrm{maxCollision}(t_{c})=0$, assign $Reward(n,t_{c})=1000$.
4. Otherwise	Iterate over all time slots t=1 to *T*. If maxCollision(t)=0, assign Reward(n,t)=−10,000. If maxCollision(t)=−3, assign Reward(n,t)=10. If maxCollision(t)=1, assign Reward(n,t)=5. If maxCollision(t)=2, assign Reward(n,t)=3. If maxCollision(t)=3, assign Reward(n,t)=1. Otherwise, assign Reward(n,t)=0.5.
5. End Loop	
6. End Algorithm	

**Table 4:** Evaluation parameters.

Parameter	Value
Number of nodes in a given cell–sector intersection	20–50–80–110–140–170–200
SF	9
SF bitrate (bps)	1757
Packet size (bit)	200
Duration (ms)	113
Channel	2

## Data Availability

The original contributions presented in this study are included in the article.
